# Relationship between internal medicine program board examination pass rates, accreditation standards, and program size

**DOI:** 10.5116/ijme.52c5.6602

**Published:** 2014-01-19

**Authors:** John L. Falcone, Jed D. Gonzalo

**Affiliations:** 1 Owensboro Health Regional Hospital, Owensboro Health Surgical Specialists, USA; 2 Medicine and Public Health Sciences, Pennsylvania State University College of Medicine, USA

**Keywords:** Certification, educational measurement, ROC curve, sensitivity and specificity, specialty boards

## Abstract

Objectives: To determine Internal Medicine residency program compliance with the Accreditation Council for Graduate Medical Education 80% pass-rate standard and the correlation between residency program size and performance on the American Board of Internal Medicine Certifying Examination.

**Methods:**

Using a cross-sectional study design from 2010-2012 American Board of Internal Medicine Certifying Examination data of all Internal Medicine residency programs, comparisons were made between program pass rates to the Accreditation Council for Graduate Medical Education pass-rate standard. To assess the correlation between program size and performance, a Spearman’s rho was calculated. To evaluate program size and its relationship to the pass-rate standard, receiver operative characteristic curves were calculated.

**Results:**

Of 372 Internal Medicine residency programs, 276 programs (74%) achieved a pass rate of ≥80%, surpassing the Accreditation Council for Graduate Medical Education minimum standard. A weak correlation was found between residency program size and pass rate for the three-year period (ρ=0.19, p<0.001). The area underneath the receiver operative characteristic curve was 0.69 (95% Confidence Interval [0.63-0.75]), suggesting programs with less than 12 examinees/year are less likely to meet the minimum Accreditation Council for Graduate Medical Education pass-rate standard (sensitivity 63.8%, specificity 60.4%, positive predictive value 82.2%, p<0.001).

**Conclusions:**

Although a majority of Internal Medicine residency programs complied with Accreditation Council for Graduate Medical Education pass-rate standards, a quarter of the programs failed to meet this requirement. Program size is positively but weakly associated with American Board of Internal Medicine Certifying Examination performance, suggesting other unidentified variables significantly contribute to program performance.

## Introduction

Upon completion of an Internal Medicine residency program, trainees must complete a certification process by the American Board of Internal Medicine (ABIM) to ensure they have met the rigorous standards of assessment and evaluation required of a practicing board-certified internist.^1^ This certification process, an assessment based upon the six competencies established by the Accreditation Council for Graduate Medical Education (ACGME), requires the completion of undergraduate and graduate education, licensure and procedural requirements, and the ABIM Certifying Examination, a multiple-choice examination designed to assess the critical thinking and medical knowledge skills possessed by the examinees. The ACGME requires individual residency programs to achieve an overall pass rate of at least 80% for first-time test takers for the most recently defined three-year period.^2^Although both individual and programmatic factors are known to influence performance on the certifying examinations, research has mainly identified individual trainee factors that are associated with examination performance. Studies in Pediatrics and Internal Medicine have shown a positive correlation between performance on the in-training service examination, conference attendance, and degree of self-directed reading of electronic resources with success on examination performance.^3^^-^^8^ However, from a residency program perspective, a relative paucity of predictors has been evaluated. In a study published over 25 years ago, the number of test-takers per program (positively) and teaching beds per resident (inversely) were shown to be correlated with composite examination scores.^9^ Only one study in Pediatrics has reported that larger programs outperform smaller programs on examination scores.^10^ The current-day information regarding the size of Internal Medicine residency programs and pass rates on the ABIM Certifying Examination is unknown.Program performance on the ABIM Certifying Examination is important for trainees and residency programs, and is crucial to the accreditation process. It is known that residency size is positively correlated with standardized test performance. The magnitude, however, is not known. Therefore, in this study we sought to determine the following: (1) Internal Medicine residency program compliance with the ACGME 80% pass-rate standard, and, (2) the relationship between residency program size and program performance on the ABIM Certifying Examination.

## Methods

### Study design

We performed a cross-sectional analysis of the most recent three-year pass rates on the ABIM Certifying Examination for all residency programs in the U.S. and Puerto Rico from 2010-2012. Electronically published data were obtained from the ABIM website, with permission to use the data granted from the Director of Research Analysis at the ABIM. This study was evaluated by the University of Pittsburgh IRB for ethical approval, and was deemed non-human-subject research.

### Data collection

Data were directly obtained from the ABIM website.^11^ Independent double-data entry was performed by the lead investigator (J.L.F.) to ensure the accuracy of extrapolated database entries. Only programs with at least 10 or more examinees over the three-year study period were included, to prevent larger magnitude of percentage changes with a smaller number of examinees.

### Data analysis

#### Compliance with the ACGME 80% pass-rate standard

Descriptive statistics were performed to compare Internal Medicine residency program performance with the ACGME 80% pass-rate standard. To assess for differences in program pass rates for Internal Medicine and Internal Medicine-Pediatrics examinees, a Chi-square test was used.

#### Relationship between program size and performance

To determine the correlation between residency program size and program performance, a Spearman’s rank correlation was used. The number of program examinees during the three-year study period was used as a representation of program size since the overall number of trainees in each program could not be identified in the ABIM database. The proportion of trainees in each program during the three-year period who passed the examination was used as the measure of program performance.

To further evaluate the relationship between program size and examination performance, receiver operating characteristic (ROC) curves were prepared. An ROC curve is a plot of the sensitivity and 1-specificity across the range of residency size cutoff points derived from 2 x 2 matrices.^12^ Each matrix compares residency program size to a cutoff (0/1) and performance comparison to the minimum ACGME 80% pass-rate standard (0/1). A nonparametric ROC curve was generated, and the cutoff of annual program size that maximized sensitivity and specificity for the ACGME 80% pass-rate standard was determined. All statistics were performed using Stata 11.1 statistical software (StataCorp, College Station, TX), using an α = 0.05.

## Results

A total of 424 training programs were included in the analysis, including 372 (87.7%) Internal Medicine residency programs (19,898 examinees) and 52 (12.3%) combined Internal Medicine-Pediatrics residency programs (756 examinees), for a combined total of 20,654 examinees. All (100%) of the programs met the inclusion criterion.

### 

#### Compliance with ACGME 80% pass-rate standard

**Figure 1:**
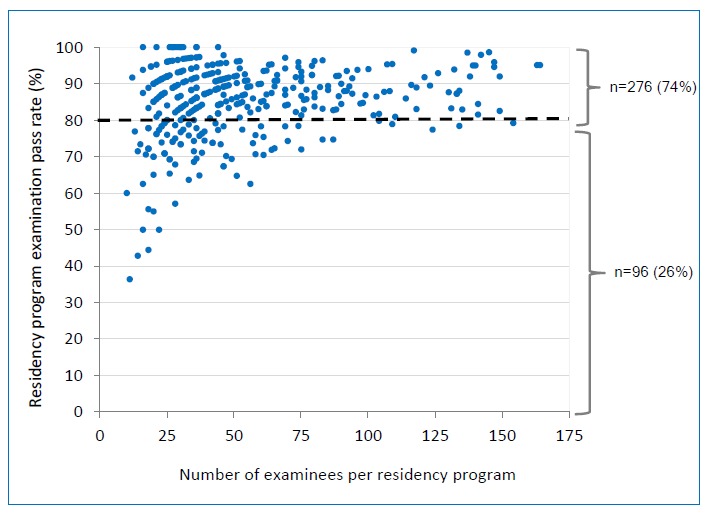
Relationship between internal medicine residency program pass rates, compliance with the ACGME 80% pass-rate standard on the American board of internal medicine certifying examination, and program size (n=372 programs).

In total, 17,867 of 20,654 examinees passed the ABIM Certifying Examination, with an overall pass rate of 86.5% There was no difference in performance between Internal Medicine examinees (pass rate = 86.5%) and combined Internal Medicine-Pediatrics examinees (pass rate = 85.7%, p = 0.52). Of the 372 Internal Medicine residency programs, 276 (74.2%) had a three-year ABIM Certifying Examination pass rate of at least 80% while 96 (25.8%) programs had a pass rate of less than 80%. The mean pass rate for Internal Medicine Residency programs was 85% ± 10%. The mean number of examinees per program during the study period was 53 ± 34. A scatter plot of program pass rates and program size with the resultant compliance with the ACGME 80% pass-rate standard is shown in Figure 1.

#### Relationship between program size and performance

**Figure 2:**
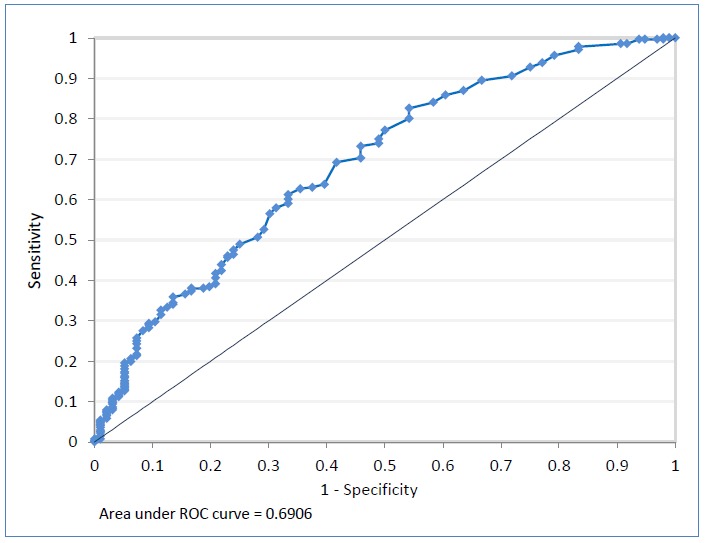
Nonparametric receiver operator characteristic curve for variable residency size cut off points in regard to residency program pass rates on the ABIM certifying examination compared with the ACGME 80% pass-rate standard.

The Spearman’s rank correlation (ρ) between program size and performance was 0.19 (p < 0.001). The ROC analysis is graphically shown in Figure 2.

There were 118 unique program sizes used in generating the nonparametric ROC curve during the study period (range [10-164]). The area underneath the nonparametric ROC curve was 0.69 (95% Confidence Interval [0.63-0.75]) (p < 0.001). The residency program size cutoff point that maximized sensitivity and specificity was 12 examinees per year. The optimal 2 x 2 matrix is shown in Table 1. The sensitivity was 63.8%, the specificity was 60.4%, the positive predictive value was 82.2%, and the negative predictive value was 36.7%. A Chi-square test showed an association between program cutoff size and performance with regard to the ACGME pass-rate standard (p < 0.001).

**Table 1:** Optimal program size that results in the highest sensitivity and specificity with comparison of program pass rate on the ABIM* CE compared to the ACGME^†^ 80% pass-rate standard of ≥ 80%.

Program Size	ACGME Standard				
		≥ 80%	<80%	Total	
≥12	176	38	214	
<12	100	58	158	
Total	176	96	372	
					

*ABIM CE: American Board of Internal Medicine Certifying Examination. ^†^ACGME: Accreditation Council for Graduate Medical Education

## Discussion

In this study investigating the relationship between the ABIM Certifying Examination with Internal Medicine residency program compliance with the ACGME 80% pass-rate standard and program size, 74% of programs were compliant and 26% were not compliant with the minimum standard. The percentage of both Internal Medicine and combined Internal Medicine-Pediatrics residency programs achieving the minimum ACGME 80% pass-rate standard is consistent with prior work in Pediatrics.^10^ To our knowledge, this is the first study to evaluate U.S. Internal Medicine residency program performance and compliance with the ACGME standard on the ABIM Certifying Examination over a recent three-year time period. Information regarding program compliance with ACGME standards on the ABIM certifying examination is vital to medical school applicants, individual trainees, and programs seeking to improve their educational and clinical environments to optimize knowledge and skills for their trainees.

We also found a weak correlation was found between ABIM Certifying Examination scores and program size, suggesting a number of additional unidentified programmatic variables are contributing to the variation in program success with the ABIM Certifying Examination. We identified an association between performance on the ABIM Certifying Examination and Internal Medicine residency program size, similar to our prior work in Pediatrics and Surgery, revealing that larger program size is associated with improved performance.^10^^,^^13^

Additionally, our results showed that programs with less than12 examinees/year are statistically less likely to meet the minimum ACGME 80% pass-rate standard. Using the area underneath the ROC results, we identified statistically significant discrimination between the ABIM certifying examination and program size in relation to the ACGME 80% pass-rate standard - this means that size can be used as an independent predictor of ABIM certifying examination pass rates. This size cut-off is identical to a recent study in Pediatrics that showed residency programs with 12 or more examinees/year satisfied the ACGME pass-rate standard.^14^ We hypothesize larger residency programs have more resources to invest in curriculum development and learning materials, recruit more highly trained faculty and educators, hire more highly-performing residents, and provide additional performance incentives, in addition to other potential confounding variables that relate program size to educational outcomes, such as examination performance.^10^^,^^13^ Although likely a primary influence on performance and a confounding variable on other unmeasured variables, program size only explains an estimated 6% of the variability in residency programs’ performance on the ABIM certifying examination. This finding enhances our overall understanding of residency program characteristics and features that may be associated with overall quality for resident performance on the ABIM certifying examination. Other variables, such as location, hospital quality, quality of teaching and educational opportunities, etc., require further exploration in subsequent studies.

These findings are important. In the context of the changing “milestone” evaluations, duty hour reform, and resultant changes in clinical care delivery models, understanding residency program factors that may be associated with high or low performance on the ABIM certifying examination are vital to improve the overall education and training of our future healthcare workforce.^15^ Although nearly three-quarters of programs were compliant with these standards, one in four ACGME-accredited programs failed to meet these standards, raising concern about the overall quality of trainees from these programs over the long term. Subsequent reactionary measures taken by the ACGME for programs failing to meet these standards can be significant, resulting in numerous strategic changes within programs to improve educational experiences for trainees.There are several limitations to our study. First, the data is limited in that the ABIM only reports pass rate data and not program completion and graduation rates. The ACGME standards also state that programs must have at least 80% of graduates take the ABIM CE in the last three years.^8^ However, despite attempts to obtain this data, they were unavailable for our analysis. Second, the analysis is cross-sectional based on retrospective data, therefore no conclusions about cause-and-effect can be drawn between ABIM certifying examination scores and program size. Lastly, we used the overall number of residents per program completing the examination as a representation of program size, which is not the most accurate measure. Further prospective research in medical education is warranted to tease out the individual and programmatic variables that are more closely associated with ABIM Certifying Examination outcomes.

## Conclusion

We found that a majority of Internal Medicine residency programs were compliant with the ACGME 80% pass-rate standard on the ABIM Certifying Examination. Larger residency programs were associated with increased pass rates and programs with less than 12 examinees per year were less likely to comply with the ACGME pass-rate standard. Although program size is associated with performance on the ABIM certifying examination, several other currently unidentified factors are contributing to high performance. Additional studies are required to identify these additional factors to inform strategies implemented by applicants, current resident trainees, and residency programs to improve the quality of performance on this examination.

## 

### Conflict of Interest

The authors declare that they have no conflict of interest.
